# Urinary Metabolites of Green Tea as Potential Markers of Colonization Resistance to Pathogenic Gut Bacteria in Mice

**DOI:** 10.20411/pai.v4i2.335

**Published:** 2019-11-14

**Authors:** Mark E. Obrenovich, George E. Jaskiw, Thriveen Sankar Chittoor Mana, Christina P. Bennett, Jennifer Cadnum, Curtis J. Donskey

**Affiliations:** 1 Pathology and Laboratory Medicine Service; Veterans Affairs Northeast Ohio Healthcare System (VANEOHS); Cleveland, Ohio; 2 Research Service; VANEOHS; Cleveland, Ohio; 3 Department of Chemistry; Case Western Reserve University; Cleveland, Ohio; 4 Department of Medicinal and Biological Chemistry; University of Toledo; Toledo, Ohio; 5 Psychiatry Service; VANEOHS; Cleveland, Ohio; 6 School of Medicine; Case Western Reserve University; Cleveland, Ohio; 7 Geriatric Research, Education and Clinical Center; VANEOHS; Cleveland, Ohio

**Keywords:** microbiota, anaerobes, mice, clindamycin, aztreonam, piperacillin/tazobactam, polyphenols

## Abstract

**Background:**

The gut microbiome (GMB) generates numerous chemicals that are absorbed systemically and excreted in urine. Antibiotics can disrupt the GMB ecosystem and weaken its resistance to colonization by enteric pathogens such as *Clostridium difficile*. If the changes in GMB composition and metabolism are sufficiently large, they can be reflected in the urinary metabo-lome. Characterizing these changes could provide a potentially valuable biomarker of the status of the GMB. While preliminary studies suggest such a possibility, the high level of data variance presents a challenge to translational applications. Since many GMB-generated chemicals are derived from the biotransformation of plant-derived dietary polyphenols, administering an oral precursor challenge should amplify GMB-dependent changes in urinary metabolites.

**Methods:**

A course of antibiotics (clindamycin, piperacillin/tazobactam, or aztreonam) was administered SC daily (days 1 and 2) to mice receiving polyphenol-rich green tea in drinking water. Urine was collected at baseline as well as days 3, 7, and 11. Levels of pyrogallol and pyrocatechol, two phenolic molecules unequivocally GMB-dependent in humans but that had not been similarly examined in mice, were quantified.

**Results:**

In confirmation of our hypothesis, differential changes in murine urinary pyrogallol levels identified the treatments (clindamycin, piperacillin/tazobactam) previously associated with a weakening of colonization resistance to *Clostridium difficile*. The changes in pyrocatechol levels did not withstand corrections for multiple comparisons.

**Conclusions:**

In the mouse, urinary pyrogallol and, in all likelihood, pyrocatechol levels, are GMB-dependent and, in combination with precursor challenge, deserve further consideration as potential metabolomic biomarkers for the health and dysbiotic vulnerability of the GMB.

## INTRODUCTION

A healthy gut microbiome (GMB) is critical to the maintenance of colonization resistance (CR), providing relative protection from overgrowth by endogenous enteric pathobionts [1, 2]. Antibiotics that reach the gut lumen can compromise CR, rendering the gut vulnerable to colonization and overgrowth by healthcare-associated pathogens such as *Clostridium difficile* and vancomycin-resistant *Enterococci* (VRE) [6–3], resulting in substantial morbidity, mortality, and economic burden [6]. Accordingly, the development of an index that reflects the health of the GMB and particularly its relative CR, would be of significant clinical value.

**Figure 1.**
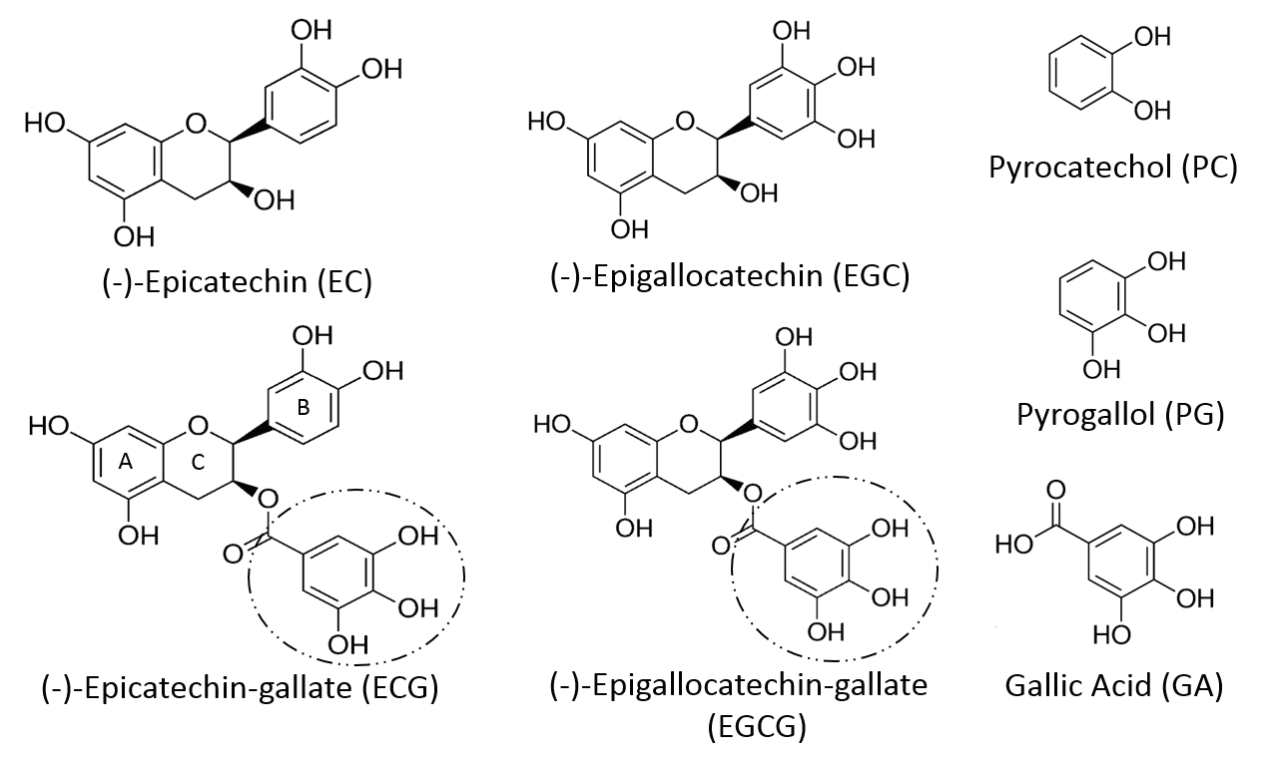
Structures of the principal flavan-3-ols in green tea ((-)-Epicatechin, (-)-Epicatechin-gallate, (-) Epigallocatechin, (-)-Epigallocatechin-gallate) and of their galloyl-derived metabolites (pyrocatechol, pyrogallol, and gallic acid). The phenolic rings (“A” and “B”) and the pyran ring (“C”) of the flavan nucleus are designated in the figure for (-)-Epicatechin-gallate. The galloylated ester moieties (circled) attached to ring “C” can be cleaved by gut microbiota.

**Supplementary Figure 1.**
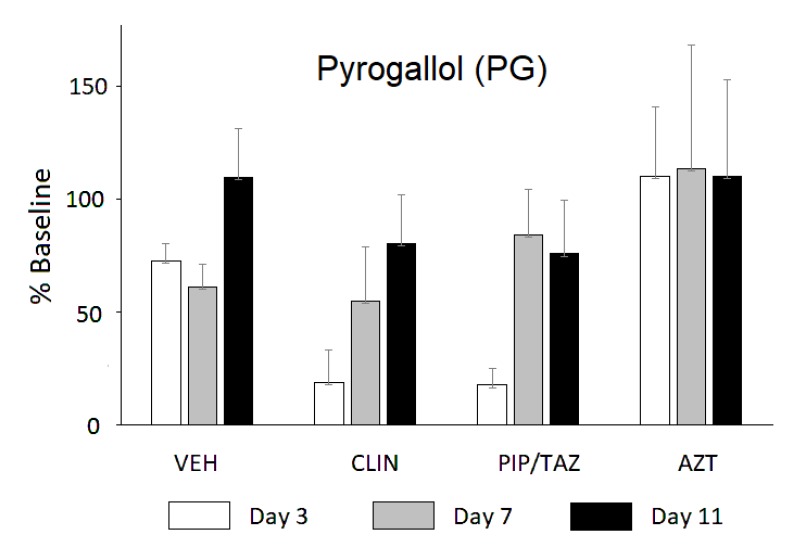
Groups of 4-5 mice were treated with clindamycin (CLIN), piperacillin/tazobactam (PIP/TAZ), or aztreonam (AZT) on days 1–2 and urine was collected on days 3, 7, and 11. Data are presented as a percent of the baseline (day 0) for the given group. Statistical analyses were conducted on the absolute levels Mean ± SEM. Urinary levels of pyrogallol (PG) tended to be lower on days 3–7 in CLIN- and PIP/TAZ-treated mice as expected but statistical significance was not reached in the data shown, due to a high level of data variance. Similar data were generated for pyrocatechol (PC).

The GMB generates numerous small molecules (< 2,000 Da) that enter the systemic circulation and are excreted in urine [9–7]. Since GMB-based processes can affect the synthesis, absorption, metabolism, and excretion of such compounds, antibiotic-induced dysbiosis may be reflected in the urinary metabolome. We initially reported that several GMB-associated urinary chemicals were significantly lower 24 hours after completion of a two-day course of antibiotics that compromises CR [10]. Subsequent studies demonstrated that urinary levels of several small phenolic molecules (SPMs) (ie, molecules MW < 2000 Da, possessing a phenolic ring) were also affected in an antibiotic-induced mouse model of dysbiosis [11]. Pyrocatechol (PC) and pyrogallol (PG) (Figure 1) are two SPMs thought to be generated through a relatively simple yet discrete pathway [12]. Both PG and PC appear in the urinary metabolome of humans, rodents, and other mammals [9, 13]. Both chemicals are unequivocally GMB-dependent in humans [12]. A PubMed search did not identify analogous studies in the mouse and only limited studies in the rat [14, 15]. Accordingly, we conducted exploratory studies on urinary PG and PC levels in antibiotic-treated mice. Although PG and PC showed the expected trends, the changes did not consistently reach statistical significance (Supplementary Figure 1). We attributed this in part to the high variance in metabolomic data [9].

In response, we considered an appropriate challenge test to amplify the changes [16]. If the syntheses of PG and PC were sufficiently elevated, the dysbiotic GMB-mediated effect on the metabolome should be easier to detect. In mammals, many SPMs, including PG and PC, can be derived from the biotransformation of polyphenols or related dietary constituents [17]. Anaerobic bacteria such as *Clostridium* species, including *C. difficile*, have been implicated in both routes [18–17]. Flavonoids constitute the largest group of dietary polyphenols [22], which despite their heterogeneity, are metabolized through a limited series of common metabolic steps in part mediated by the GMB [17, 23]. Indeed, the administration of flavonoids has been used to delineate such pathways [17, 24]. Green tea is a particularly attractive candidate in this regard. Its composition and metabolism have been well characterized. Four catechins, (-)-epicatechin (EC), (-)-epigallocatechin (EGC), (-)-epicatechin-3-*O*-gallate (ECG), and (-)- epigallocatechin-3-*O*-gal-late (EGCG) (Figure 1) together comprise over 92% of all the flavonoids in brewed green tea [25] (Supplementary Table 1).

About one-third of ingested catechins are metabolized and absorbed in the small intestine. From there they enter the portal circulatory system and become vulnerable to hepatic metabolism and then fecal and/or urinary excretion. The remaining two-thirds reach the colon, where they can undergo bacterially mediated biotransformation (eg, dehydroxylation, demethylation) and then be metabolized into compounds that are either excreted in feces or absorbed systemically and excreted in urine [12, 24–26]. The bioavailability of green tea catechins is comparable in humans and in the mouse [29]. Antibiotic-treated mice have elevated plasma, serum, and tissue levels of several parent catechins, presumably due to inhibition of GMB-mediated biotransformation [30]. GT ingestion elevates urinary levels of PG and PC in individuals with an intact colon, but not in those with an ileostomy [12, 31], strongly implicating the GMB. Accordingly, we tested the hypothesis that antibiotic treatments previously shown to lower other SPM levels [10, 11], would potently suppress GT-augmented urinary PG and PC levels in the mouse.

## MATERIALS AND METHODS

### Animals

The study protocol was approved by the Cleveland VA Medical Center's Institutional Animal Care and Use Committee. Female CF-1 mice weighing 25–30 g (Harlan Sprague-Dawley, Indianapolis, IN) were housed 4-5 / cage with groups segregated by antibiotic treatment. All cages were in the same rack and subjected to the same husbandry in order to minimize cage effects. They were fed a sterilized Teklad Global 18% protein-extruded rodent diet (Harlan Teklad, Madison, WI) based on plant products, including soybean meal, brewer's dry yeast, and wheat [32].

### Treatment

Fresh (GT) solution was prepared twice daily (8:00H and 17:00H) to coincide with the start of the lights on/off periods. One green tea bag (Kirkland®) was steeped for 3 min in 200 ml double-distilled water and then cooled before use. No inhibitors, preservatives, or metal chelators were added to the GT solution. In earlier studies, bacterial cultures were not found to appreciably affect end-product formation from tea (data not shown) [33].

Mice were conditioned to drink increasing concentrations of GT (increase by 20%/d) over one week preceding the start of antibiotic treatment, so that the target concentration ~(0.682 green tea catechin content [34]) was reached before and maintained through the period (days 0–11) of antibiotic treatment and urine collection. On days 1 and 2, groups (n = 5–8 mice/group) received subcutaneous (SC) injections (once daily at 11 a.m.; 0.1 ml total volume) of either saline (SAL) or one of three antibiotic regimens: (1) clindamycin (CLIN) (15 mg/day); (2) piperacillin/tazobactam (PIP/TAZ) (8 mg/day); or (3) aztreonam (AZT) (3 mg/day). The antibiotic doses were selected to model typical clinical doses (mg/g body weight over 24 hours). CLIN and PIP/TAZ inhibit intestinal anaerobes, thus promoting the growth of *C. difficile* or other pathogens; AZT targets facultative gram-negative microorganisms but not anaerobes and does not promote pathogen colonization [3–5]. One group of mice did not receive either GT or antibiotic and acted as an additional control. Baseline urine samples for analysis were collected on day 1 (immediately preceding the first administration of antibiotics) and the collection repeated on days 3, 7, and 11.

### Sample Acquisition

On the morning of days 1 (pre-antibiotic baseline), 3, 7, and 11, mice were placed individually in clean, food-free, and bedding-free cages with Hydrogel® (for hydration) and observed continuously for one-hour periods until they produced ~100μL urine. Fresh urine specimens (100 μL aliquots) were collected in real time by pipette tip and transferred to sterile 1.5 ml Eppendorf tubes on wet ice until the end of the collection.

Stool and any urine in contact with stool was removed and the floor wiped continuously during the observation period. In addition, three types of controls were employed to rule out spurious contamination: (1) 100 μL (total) of sterile double-distilled water was placed across three different points on the floor of a clean individual cage and collected after 60 min; (2) 100 μL (total) of double-distilled water was placed across three different points on the floor and a mouse immediately introduced. Feces and urine were continuously removed over the following 60 min. The remaining water was then immediately collected and, (3) a mouse was placed into a clean cage. After 60 min both the mouse and all visible urine and feces were removed. 100 μL (total) of double-distilled water was placed across three different points on the floor and collected after an additional 60 min had elapsed. All specimens were quick-frozen on dry ice and then stored at –80°C until they were thawed for analysis.

### Chemicals and Reagents

Antibiotics were USP grade (Pfizer, New York). All chemicals and reagents were of the highest purity and grade commercially available. Standards were purchased from Sigma-Aldrich Co. LLC. (St. Louis, MO). LC-MS grade solvents: water; acetonitrile (ACN); formic acid; and methanol were purchased from Fisher Scientific (Pittsburgh, PA).

### Preparation of Standards

A standard stock solution containing all of the compounds of interest as well as the internal standards (1 mg/ml) was prepared in a suitable solvent (LC/MS grade water or methanol), initially blanketed with nitrogen gas, in glass vials equipped with Teflon®-lined screw caps and then stored at –20°C. The thawed stock solution was serially diluted to obtain work solutions in the range of 10-1000 ng/ml. To generate standard curves for quantitation of the metabolites, stripped urine was used as the matrix. In brief, 10 μL of the internal standard (1000 ng/ml) and 50 μL of stripped urine were added to each serially diluted standard, which was then brought to a final volume of 100 μL.

### Urine Sample Preparation for LCMS

Thawed samples were centrifuged (13,000 g × 15 min at 8°C), hydrolyzed (β-glucuronidase and aryl sulfatase) for 24 h at 37°C per the manufacturer's instructions (Roche Diagnostics GmbH, Mannheim, Germany). 10 μL of multiple internal standard were added to 100 μL of rodent urine [35]. The vials were either immediately processed or were weighed and refrozen at –80°C. Immediately prior to analysis, 500 μL of ice-cold 100% methanol were added to precipitate any possible protein. The sample was vortexed for 20 seconds; the supernatant was transferred to a clean 1.5-mL Eppendorf tube. It was brought to near-dryness using a Speed-Vac® centrifuge and then reconstituted with 100 μL of solvent (water + 0.01% formic acid),

### Compound Identification and Metabolite Analysis

Aliquots of 2 μL were placed in the autosampler (8°C). The analytic Shimadzu HPLC system (Shimadzu Kyoto, Japan) parameters were: Luna, C18 (3 um, ODS 100A, 2 × 150 mm) LC column (Phenomenex, Rancho Palos Verdes, CA) with an integrated guard column to minimize and/or eliminate cross-contamination by sample matrix between sample runs. Water injections were conducted with every 20 samples to monitor for possible carryover. Chromatography was performed at a constant flow of 0.3mL/min with the following parameters: nebulizing gas flow 3 L/min, heating gas flow 10 L/min, drying gas flow 10 L/min, interface temperature 300°C, DL temperature 250°C, heat block temperature 400°C, argon as the collision-induced dissociation (CID) gas. Eluents A (water + 0.01% formic acid) and B (acetonitrile + 0.01% formic acid) were used under gradient conditions at a flow rate of 0.3 mL/min. Isocratic elution with 100% A (0-2 min, followed by 100% A to 100% B; 2-8 min; 8-18 min, 100% B, 18-18.5 min, 100% B to 100% A; 18.5-26 min, 100% A) achieved separation of PG and PC. Second, 5μL of HPLC column effluent was introduced onto a Shimadzu 8050 triple quadruple MS system (Shimadzu, Kyoto, Japan) and analyzed using either positive or negative electrospray ionization (ESI) in the MRM mode. The data were analyzed using Liquid Chromatography Mass Spectrometry (LC/MS) software (LabSolutions®, Shimadzu, Kyoto, Japan). Levels of pyrogallol (PG) and pyrocatechol (PC) were measured.

Resulting data files were exported to our servers. The data were processed manually to authenticate metabolite spectra using retention index and mass spectrum information. Identified metabolites were reported if present in all of the samples (as defined in our method); the peak area ratios (unique ion/internal standard) were used for quantitation [10]. All values were within the limits of detection for the assay. Missing data were identified and replaced from raw data set files.

### Statistical Analysis

At each time point, levels of metabolites were subjected to a one-way ANOVA followed by pairwise comparisons with Bonferroni corrections. Because mice within treatment groups were not individually tracked through successive urine collections, a repeated measures analysis was not applicable. The significance level was set at *P* < 0.05.

## RESULTS

**Figure 2.**
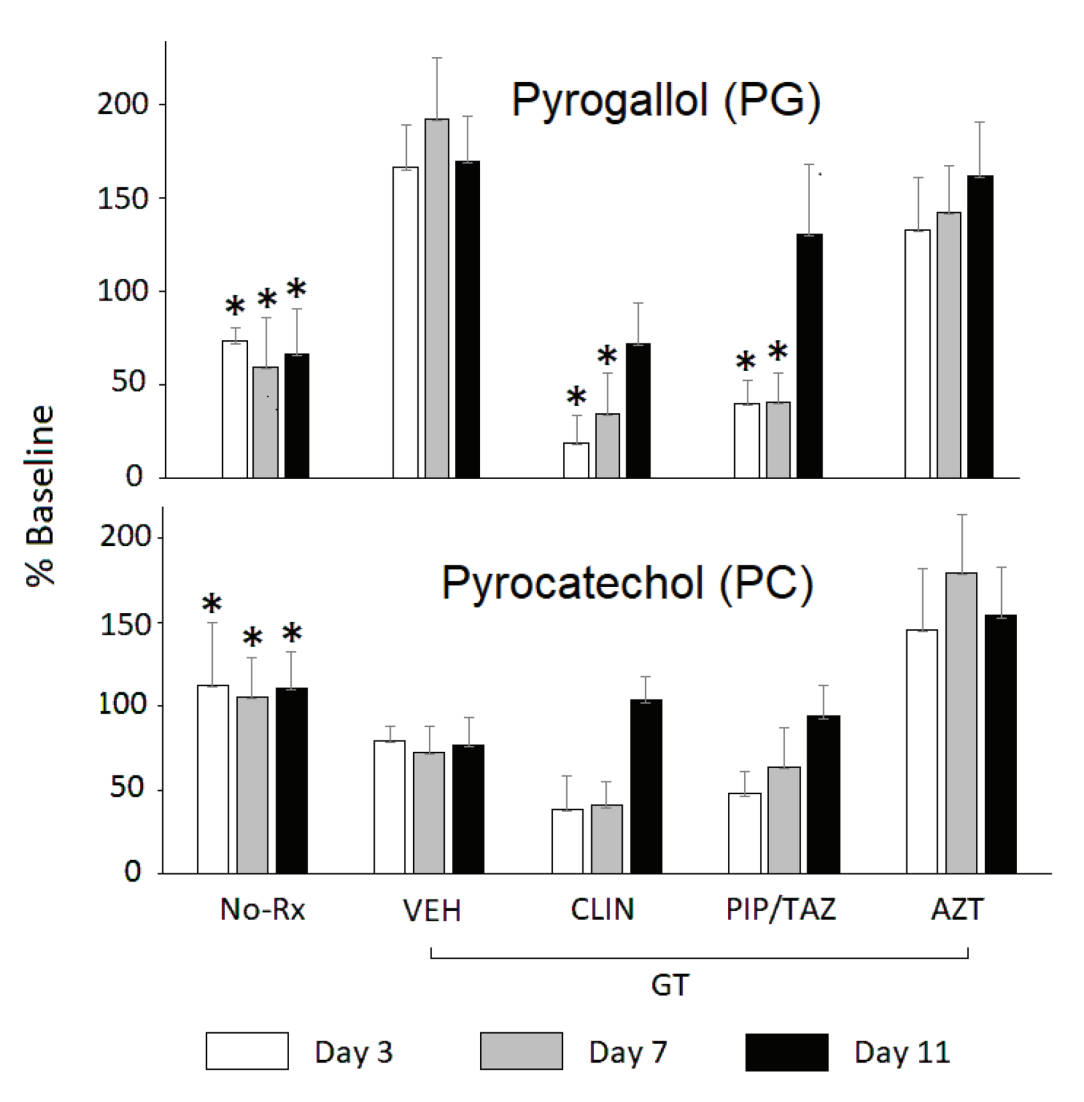
One group of mice (No-Rx) did not receive either green tea (GT) or antibiotics. Of the remaining groups, all received GT in drinking water continuously and in addition either a saline vehicle (VEH), clindamycin (CLIN), piperacillin/tazobactam (PIP/TAZ), or aztreonam (AZT) SC on days 1–2. Data are presented as a percent of the baseline (day 0) for the given group. Statistical analyses were conducted on the absolute levels. Mean + SEM. *significantly different from VEH (ie, GT only) for the corresponding day, corrected for multiple comparisons.

At baseline (day 0), that is, prior to the start of antibiotics, there were no significant differences in PG or PC levels across the treatment groups (Table 1, Figure 2). For PG, the overall ANOVA for PG day 3 showed a treatment effect (F(4,18) = 8.93, *P* < 0.0004). Bonferroni-adjusted pairwise comparisons showed that levels in mice treated with GT only were significantly different from those that in addition received CLIN (*P* < 0.01), PIP/T (*P* < 0.01) or no treatment at all (*P* < 0.01). In addition, PG levels in mice treated with AZT were significantly higher than those treated with CLIN (*P* < 0.05) or in the No-Rx group (*P* < 0.05) (Figure 2). The overall ANOVA for day 7 showed a treatment effect (F(4,20) = 7.59, *P* < 0.0007. Levels in mice treated with GT only were significantly different from those treated with CLIN (*P* < 0.04), PIP/TAZ (*P* < 0.01) or not treated at all (*P* < 0.01). AZT treated mice had higher levels than those that were not treated at all (*P* < 0.05). The overall ANOVA for day 11 showed a significant treatment effect (F(4,20) = 3.85, *P* < 0.02). Significance was confined to the pairwise comparisons between the groups no-Rx vs GT (*P* < 0.05) and no-Rx vs AZT (*P* < 0.05).

**Table 1.** 

ng/ml	No-Rx VEH	Green Tea (GT)			
		VEH	CLIN	PIP/TAZ	AZT
PG	94.98 ± 27.04	324.00 ± 111.62	470.19 ± 144.43	305.65 ± 123.74	325.58 ± 95.03
PC	20.78 ± 3.16	352.64 ± 76.24	234.11 ± 38.79	335.53 ± 86.98	311.57 ± 61.02

Baseline (day 0) urinary levels of pyrogallol (PG) and pyrocatechol (PC) in mice prior to the introduction of antibiotics. One group of mice (No-Rx) did not receive either green tea (GT) or antibiotics. Of the remaining groups, all received GT in drinking water and were assigned to additional treatment with either a saline vehicle (VEH), clindamycin (CLIN), piperacillin/tazobactam (PIP/TAZ), or aztreonam (AZT) on days 1–2. Mean ± SEM.

For PC, the overall ANOVA for day 3 showed an overall effect of treatment (F(4,25) = 7.95, *P* < 0.0003). Bonferroni-adjusted pairwise comparisons showed that levels in mice treated only with GT were significantly different from those in mice that had no treatment at all (*P* < 0.01) (Figure 2). In addition, PC levels in mice treated with AZT were significantly higher than those not treated at all (*P* < 0.01) or those treated with CLIN (*P* < 0.01) or PIP/TAZ (*P* < 0.01). The overall ANOVA for day 7 showed an overall effect of treatment (F(4,25) = 9.77, *P* < 0.00007. Levels in mice treated with GT only were significantly different from those not treated at all (*P* < 0.05). AZT treated mice had higher levels than those not treated at all (*P* < 0.01), or those treated with CLIN (*P* < 0.01) or PIP/TAZ (*P* < 0.01). The overall ANOVA for day 11 showed a significant treatment effect (F(4,22) = 7.94, *P* < 0.0004) that was evident in the pairwise comparisons between the groups no-Rx vs CLIN (*P* < 0.05) and no-Rx vs PIP/TAZ (*P* < 0.01). On each of days 3 and 7, PC levels in the GT group were significantly different from those in the CLIN group, as determined by uncorrected t-test. Trends for differences between mice treated with VEH and those treated with either AZT or PIP/TAZ did not withstand the correction for multiple comparisons.

## DISCUSSION

After administration of polyphenol precursors, antibiotic effects on urinary levels of PG were clearly evident (Figure 2, Table 1). Furthermore, urinary PG levels were *differentially* affected by antibiotics previously shown to suppress versus not suppress intestinal anaerobes and promote colonization by healthcare-associated pathogens [3–5]. Specifically, CLIN and PIP/TAZ suppress anaerobes; treatment with these agents significantly lowered urinary PG levels. In contrast, treatment with AZT, which has little if any impact on anaerobes, did not lower levels of PG. The trends were similar but did not reach rigorous statistical significance in the case of PC. The data must be interpreted in light of experimental limitations and the multiple processes that can affect constituents of the urinary metabolome.

## GMB-MEDIATED METABOLISM OF CATECHINS

In the rat, orally administered EC is extensively glucuronidated and methylated by enterocytes of the small intestine during absorption, with the result that little free EC enters the portal circulation [22, 36, 37]. The compound and metabolites then undergo hepatic sulfation and are additionally exposed to catechol-*O*-methyl transferase in the liver and kidney [37, 38]. Major phase II conjugation and metabolism processes include methylation; β-glucuronidation of hydroxyl-, carboxyl-, amino-, and thiol-groups; as well as sulfation of hydroxyl- and amino groups [17, 39]. In the rat, 18 different mono-, di-, and triconjugates of EC with various glucuronide, methyl, and sulfate moieties have been identified [40]. A fraction of those metabolites enter the systemic circulation and are renally excreted. The remainder enter bile where their levels peak within 2h of oral administration of the parent [41]. After reaching the small intestinal lumen, some compounds re-enter the enterohepatic circulation, while the rest are exposed to the GMB, which increase in density and diversity through the lower ileum and large intestine [17, 42].

Catechins share a common flavan structure consisting of two phenol rings (A and B) joined by a heterocyclic pyran ring (C) (Figure 1) [43, 44]. While no endogenous mammalian enzymes are known to cleave the “C” ring [13], mixed [17] as well as certain individual GMB constituents (eg, in the rat, *Adlercreutzia equolifaciens, Eggerthella lenta, Flavonifractor plauti*) [45] can do so. *In vivo* tracer studies using radio-labeled catechin demonstrate that, across species, (rat, guinea-pig, humans) the “B” ring is maintained within subsequent metabolites [48–46], with the exception of those chemicals derived exclusively from the galloyl moiety present in some members of the catechin family. Indeed, ECG and EGCG differ from EC and EGC in having an ester-linked galloyl moiety (Figure 1) which can be readily cleaved in *in vivo* to liberate free gallic acid (GA) [49]. Ingestion of foods or extracts containing either free or esterified GA [50, 51] elevates uri-nary levels of GA and its metabolites [53–52]. As far as GT is concerned, ECG and EGCG are the principal flavonoids and dominant sources of GA (Supplementary Table 1). The GA contribution from gallocatechin-gallate is negligible [12].

In mice receiving GT, broad-spectrum antibiotic-induced suppression of the GMB results in elevated serum and fecal levels of EGCG but lowered fecal levels of EGC, presumably due to such attenuated hydrolysis of the galloyl ester bond in EGCG [30]. Administration of theaflavin digallate, another dietary flavonoid esterified with gallate, elevates fecal excretion of GA levels in normal but not in germ-free mice [55]. Cultures of porcine GMB can generate GA from ECG or EGCG but not from EC or EGC [56]. Analogously, human fecal slurries or even individual *Bifidobacterium* strains can hydrolyze ECG or theaflavin digallate to generate GA [55, 57]. The single report that galloylated catechins are resistant to hydrolysis by cultures of rat feces [57] is difficult to reconcile with the demonstration that several bacterial strains isolated from rat GMB (*Enterobacter aerogenes, Raoultella planticola, Klebsiella pneumoniae* subsp*. pneumoniae*, *Bifido-bacterium longum* subsp. *Infantis)* hydrolyze EGCG into the constituent EGC and GA [58]. Thus, as a general rule, the mammalian GMB appears to possess the capacity to hydrolyze galloylated flavonoids to generate free GA.

Free GA is then subject to additional biotransformations. In the rat, the rabbit, and in humans, orally administered GA is readily metabolized to urinary 4-*O*-methyl-GA [49, 59, 60] as well as other metabolites [61]. Gavage with ECG or GA elevates urinary PG and/or its metabolites in the rat and rabbit, consistent with a simple decarboxylation of GA to PG [14, 49, 61, 62]. Two reports in the rat indicate that at least some of these steps can be suppressed by broad-spectrum antibiotics targeting the GMB [14, 15]. Analogous studies in the mouse are lacking but human data are more abundant.

**Figure 3.**
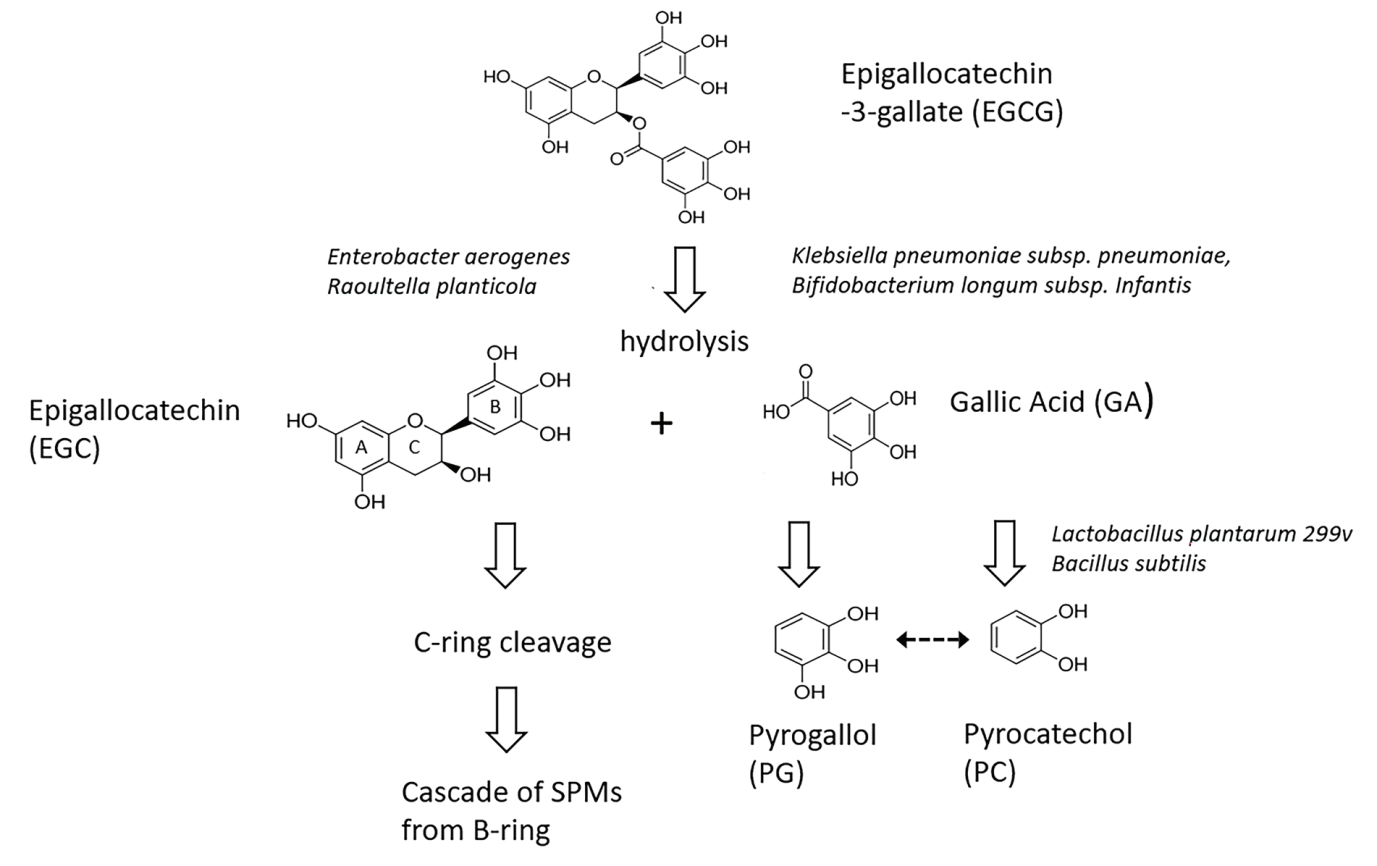
Possible metabolic pathways from (-)-epigallocatechin gallate to pyrogallol and pyrocatechol. Known bacterial constituents of the GMB mediating a given step are italicized. Possible interconversions of pyrogallol and pyrocatechol (dotted arrow) are less well characterized. SPMs: small phenolic molecules.

Foods rich in free GA and/or galloyl-esterified flavonoids elevate excretion of PG and PC or their conjugates in healthy controls (Figure 1) [31, 63–63]. Ingestion of GT or grape juice elevates excretion of PG and/or PC in patients with an intact colon, but not in those with an ileostomy [12, 66]. While cultures of the GMB colonizing the remaining ileum can dehydroxylate GA to PG *in vitro* [67] this does not appear to be sufficient to affect urinary PG levels *in vivo*. Human fecal cultures are able to generate PC and/or PG from sources of GA or galloylated esters [12, 55, 57, 66, 67–68]. Several identified bacterial strains (*Lactobacillus plantarum 299v, Bacillus subtilis*) can decarboxylate GA to PG on their own [55, 72]. While the underlying reactions, and particularly the relationship between PG and PC remain to be fully characterized, the available data suggest a provisional pathway (Figure 3). [45]

## ANTIBIOTIC EFFECTS

The data suggest that anaerobic bacteria, differentially suppressed by CLIN or PIP/TAZ vs AZT, are critically involved in generating PG and PC from the galloylated catechins. This suppression could act at any of several loci: (1) hydrolysis of EGCG and EG to release GA; (2) decarboxylation of GA to generate PG; or (3) possible interconversions of PC and PG (Figure 3). Most bacterial enzymatic capacities are expressed by multiple types of bacteria rather than being exclusive to a single genus [73]. Thus, the list of individual strains identified in the pathways of interest so far (Figure 3) [55, 58] should be considered preliminary. Given the diversity of reactions expressed in the GMB *in vivo* [73], additional pathways that generate PG or PC are certainly possible [74]. Nonetheless, we can conclude that an oral precursor rich in galloylated catechins markedly elevates urinary levels of PG in the mouse through a GMB-dependent mechanism.

## VARIANCE AND EXPERIMENTAL LIMITATIONS

Although the trends for PC were very similar to those for PG, the lowered PC levels did not reach statistical significance when a rigorous correction for multiple comparisons was imposed (Figure 2). By our estimate, had the number of mice per cell been doubled, the suppression of PC levels would have been significant. Similarly, had individual mice been tracked across sample collections, a repeated-measures analysis with its greater power could have been applied. Given translational considerations, we were seeking a highly robust index that would not depend on either large numbers of samples or conservative statistical methods to achieve significance. However, even with a precursor load that elevated PC and PG levels by up to 10-fold, substantial variance remained. Diet and the microbiome are major sources of such variance [17].

Our mice were fed a largely plant-based laboratory diet consisting of soybean meal, brewer's dry yeast, and wheat [32]. Soybeans contain only low quantities of catechin and cinnamic acids [75] and brewer's yeast does not contain polyphenolic precursors of PAs. Wheat and most other grains do, however, contain large amounts of ferulic acid [76, 77]. Ferulic acid, however, is not known to be converted *in vivo* to either PG or PC. Thus, the galloylated catechins in GT should constitute the dominant source of precursors for urinary PG and PC in our study.

We used an outbred mouse strain that is more robust, but also more microbially diverse than inbred strains [78]. Even in co-housed mice of the same strain, significant inter-individual variations in the effects of antibiotics on the GMB are evident [79]. We previously reported on GMB changes induced by common antibiotic regimens [5] but did not do so in this study. Accordingly, we cannot determine possible correlations between bacterial subtypes and the declines in PG or PC.

Both PC and PG have multiple conjugates. In humans, for instance, 9 urinary conjugates of PG have been identified after GT ingestion [80]. Most such chemicals are not commercially available for use as authentic standards. Accordingly, in the current study we hydrolyzed urine samples using both β-glucuronidase and aryl sulfatase before chemical analysis. The hydrolysis liberated PG and PC from glucuronidated and/or sulfated conjugates but not from methylated conjugates. We cannot preclude the possibility that one or another of these conjugates would have provided a more robust index of antibiotic effects. We observed that levels of PG in the animals treated only with green tea were non-significantly elevated (~150%) relative to their pretreatment baseline (Figure 2). This may be attributable to the initial continuous rise in plasma catechins during administration of tea polyphenols to rodents [81].

Since flavonoids and their metabolites undergo hepatic biotransformation [17], changes in hepatic function can also affect their kinetics. Usual doses of most antibiotics, however, generally have little effect on hepatic function [82]. While high IV or IP doses of PG can inhibit catechol-*O*-methyl-transferase [83] as well as sulfation [84], whether PG levels (< 8 μM in the rat) generated by oral precursors [85] possess meaningful bioactivity is not known. Finally, we evaluated only female mice, in keeping with the larger body of work from our laboratory (CJD). Extrapolation of our findings to male mice should be made cautiously in light of potential gender-specific effects of antibiotics on the GMB [86].

## TRANSLATIONAL IMPLICATIONS AND CONCLUSIONS

Our group had previously demonstrated that treatment with CLIN or PIP/TAZ weakened CR to overgrowth by *C. difficile* [6–3]. Evaluation of the corresponding urinary metabolome showed suppressed levels of 3,2-hydroxyphenylpropionic acid (3,2-HPPA) as well as of 3,3-HPPA and 3,4-HPPA, SPMs known to be at least partially derived by the GMB through C-ring cleavage of dietary flavonoids (Figure 3) [11]. Our current data expand on this, indicating that urinary PG, more robustly than PC, is also lower in association with compromised CR. Several next steps are possible. Given that both PG and PC are derived from GA, the administration of the purely galloylated moieties ECG and EGCG or even pure GA could be considered for precursor loading. One risk with GA loading is that this chemical itself possesses antibacterial activity [87] and, at higher concentrations, can inhibit its own GMB-mediated metabolism [56]. A similar effect *in vivo* cannot be precluded. Another approach would be to administer GT and to simultaneously measure both those SPMs derived from C-ring cleavage as well as those derived from hydrolysis of galloylated catechins (Figure 3). The results could suggest a broader biomarker panel.

We conclude that urinary PG and PC levels in the mouse are GMB-dependent, and that this dependence may be amplified by precursor loading. Such an effect may be even larger in humans, in whom the relative increase in urinary PG levels after GT ingestion exceeds those of any other metabolite [31]. The data linking PG and PC levels in humans to the GMB are particularly robust [12, 55, 57, 66, 67–68]. Our results build on earlier studies in which oral administration of flavonoid-rich dietary constituents, whether in the form of natural food products (eg, green tea, coffee, fruit juice) or as purified supplements (eg, catechin, quercetin) was used to delineate metabolism of polyphenols in rodents and humans [31, 47, 48, 59, 64]. Insofar as steps in that metabolism necessarily involve the GMB, appropriate precursor loading may facilitate development of clinically useful indices, including the degree of vulnerability to overgrowth by pathogenic anaerobic bacteria. Since both PC and PG reach the central nervous system [88] and can affect cerebral monoamine levels [89], possible neuropsychiatric effects should also be explored.

## Figures and Tables

**Supplementary Figure 2.**
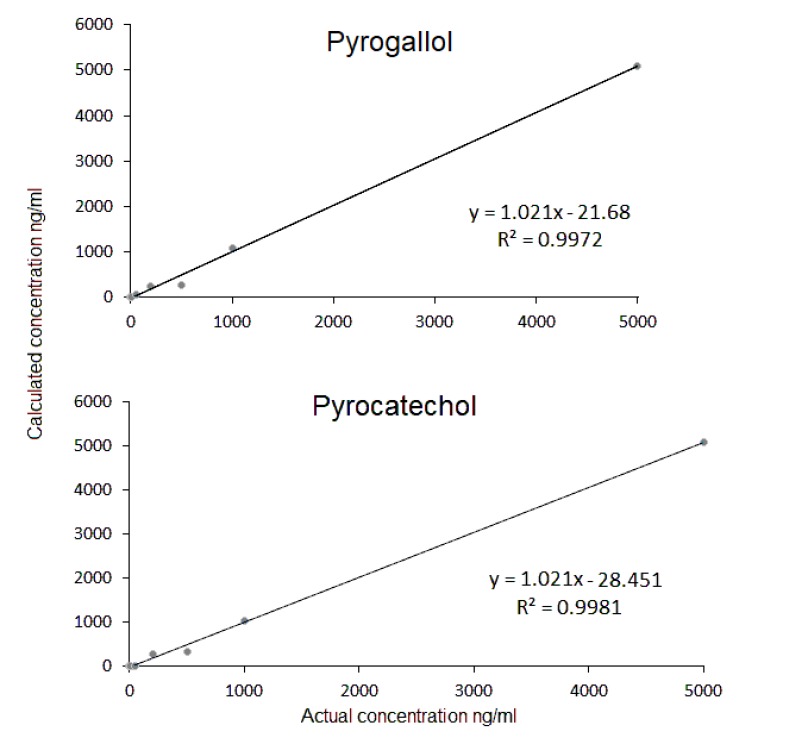
Calibration curves for the compounds of interest.

## SUPPLEMENTARY MATERIALS

**Supplementary Table 1.** Derived from USDA data.

Constituents of brewed green tea	% total flavonoids	mg/100g
(-)-Epicatechin	6.22	8.29
(-)-Epicatechin-3-*O*-gallate	14.8	19.73
(-)-Epigallocatechin	12.5	16.71
(-)-Epigallocatechin-3-*O*-gallate	58.4	77.81
Subtotal	91.92%	122.54

**Supplementary Table 2.** MRM transitions for compounds of interest as well as internal standards.

Compound	ESI Mode	MRM Transition (m/z)	RT (min)
Gallic acid	(-)	169.00>125.15	5.156
Pyrogallol	(-)	125.10>79.10	5.154
Pyrocatechol	(-)	109.10>91.10	5.994
d5-L-phenylalanine	(-)	169.00>152.20	5.141
